# Physical activity modifies the association of the composite dietary antioxidant index with all-cause mortality in the US osteoarthritis population

**DOI:** 10.3389/fpubh.2023.1297245

**Published:** 2023-12-04

**Authors:** Yiwei Zhang, Zhengwei Duan, Hengli Lu, Guanghua Lu, Yuesong Fu, Guodong Li, Sen Wang

**Affiliations:** Department of Orthopedics, Shanghai Tenth People's Hospital, Tongji University School of Medicine, Shanghai, China

**Keywords:** composite dietary antioxidant index, osteoarthritis, physical activity, mortality, interaction

## Abstract

**Background:**

It remains unclear how antioxidant intake affects all-cause mortality in osteoarthritis (OA) patients. In this prospective cohort study, we aim to explore the association of the Composite Dietary Antioxidant Index (CDAI) with all-cause mortality and investigate the interaction of physical activity (PA) and CDAI on all-cause mortality in OA populations.

**Methods:**

A total of 3,197 adults with OA in the National Health and Nutrition Examination Survey (NHANES) from 2001 to 2018 were included in this study. Death outcomes were obtained from National Death Index (NDI) records. Multivariable Cox regression analyses with cubic spines were applied to estimate the association of CDAI with all-cause mortality. The interaction between CDAI and PA on all-cause mortality was further assessed in stratified analysis and interaction tests.

**Results:**

The hazard ratios for all-cause mortality were 0.95 (0.77–1.17) for Q2, 0.75 (0.59–0.97) for Q3, and 0.71 (0.55–0.92) for Q4 (*P* for trend <0.001), compared with the lowest quartile of CDAI. A negative linear association was found between CDAI and all-cause mortality. In the stratified analyses, CDAI was negatively associated with all-cause mortality in the insufficient PA group. While in the low and sufficient PA group, there were nonlinear relationships of CDAI with all-cause mortality.

**Conclusion:**

A negative linear relationship was observed between CDAI and all-cause mortality in OA patients, and this association was significantly modified by PA. Higher intake of dietary antioxidants might be the interventional objective to reduce the risk of all-cause mortality in the US OA population.

## Introduction

Osteoarthritis (OA) is a degenerative joint disease and characterized by persistent pain, chronic inflammation, and functional limitations (1). It is prevalent in middle-aged and aged people. Statistically, 9.6% of men and 18.0% of women over 60 have symptomatic OA (2). At least 130 million individuals may suffer from OA by the year 2050 (2), resulting in a societal burden. The immobility caused by the OA has greatly changed the lifestyle of patients and caused many comorbidities, such as cardiovascular and metabolic diseases, which lead to an increased risk of mortality in OA patients (3).

Various factors activate chondrocytes, induce cartilage destruction and further cause cartilage degeneration may be the primary pathogenesis of OA (4). Among these factors, oxidative stress, caused by aberrant reactive oxygen species generation that disrupts chondrocyte redox homeostasis, has been demonstrated to be a crucial factor in the pathogenesis of osteoarthritis (5, 6). Previous studies have reported that dietary antioxidant has a significant influence on the development and progression of OA, making it a great candidate for synergistic treatment (7–9). However, it remains unclear how dietary antioxidants affect the prognosis of OA individuals.

The composite dietary antioxidant index (CDAI) measures an individual’s antioxidant profile by considering dietary antioxidants such as vitamins A, C, E, selenium, zinc, and carotenoids (10). It has been found that stroke patients with higher CDAI have a lower risk of all-cause mortality (11). However, similar efforts among the OA population have not yet been conducted. Furthermore, physical activity (PA), which was considered the first-line non-pharmacological treatment for OA, can significantly reduce pain and activity limitations in OA patients (12). Accumulated evidence has proved the efficacy of PA in decreasing the risk of mortality (13, 14). Since both CDAI and PA can individually affect the prognosis of individuals, it is uncertain if CDAI and PA have an interaction on mortality in patients with OA. In this study, based on the National Health and Nutrition Examination Survey (NHANES), we investigated and assessed the relationship between CDAI, PA and mortality among OA patients.

## Methods

### Data source and population

Data were gathered from the NHANES, a nationally representative, periodical cross-sectional sample survey for the health and nutrition of the non-institutionalized civilian population in the United States. Detailed survey methods and processes were approved by the National Center for Health Statistics (NCHS) and are available on the NHANES website.^1^ Consents were obtained in writing from all participants. Over nine survey cycles from 2001 to 2018, NHANES enrolled 91,351 participants over 20 years old, 4,911 of them had been diagnosed with OA. After excluding samples with missing dietary data (*n* = 513), mortality status (*n* = 9), covariate data less than 10% missing, and the 5th and 95th percentile of CDAI, to avoid the effect of outliers due to recall bias, 3,197 patients with OA were included in our final study.

### Exposure information

24-h dietary recall interview of NHANES, conducted by professional diet interviewers at the Mobile Examination Center (MEC), aims to collect e details of participants’ dietary consumption in the 24 h prior to the interview. To evaluate the influence of daily dietary antioxidant intake on mortality in US populations with OA, we calculated the CDAI proposed by Wright et al. The detailed calculation method has been described in previous studies (10). To put it briefly, standardization of dietary intakes on the six antioxidants was performed by minus the average value and dividing by the standard deviation. Following that, the standardized dietary antioxidant intakes were added up to determine the CDAI. We used the Global Physical Activity Questionnaire to investigate the PA status of participants (15). Following the World Health Organization (WHO) recommendations, the weekly average in minutes of moderate to high-intensity PA, frequency, and metabolic equivalent (MET) value recommended by NHANES were multiplied to obtain the metabolic equivalent of weekly PA (MET-min/wk) (16). All OA patients included were separated into the low PA group (PA = 0), insufficient PA group (< 600 MET-min/wk), and sufficient PA group (≥ 600 MET-min/wk).

### Ascertainment of mortality outcome

The death status of OA patients in this study cohort was derived from death certificate records in the National Death Index which was linked with NHANES by probabilistic match. All-cause mortality was determined as death for any cause, and cardiovascular disease (CVD) mortality was defined according to the International Classification of Diseases, 10th Revision (17).

### Assessment of covariates

Covariates were selected based on previous studies and recommendations of clinical experts (18). Demographic information was collected by trained interviewers through standardized questionnaires, including age, gender, education level, ethnicity, marital status, poverty income ratio, which was calculated according to the Department of Health and Human Services (HHS) poverty guidelines, history of the disease (hypertension, diabetes), alcohol use, and prescription medicine taken. In particular, participants who consume ≥12 drinks per year alcohol drinking are defined as drinkers and < 12 drinks are defined as non-drinkers (19). Participants were classified as having a history of diabetes or hypertension if they had received a diagnosis of the condition from a doctor or were taking medication prescribed to treat it. Body mass index (BMI, < 25 kg/m^2^, ≥ 25 kg/m^2^) was measured in the MEC. Serum cotinine was measured in the laboratory and divided into exposed and non-exposed groups due to a cut-off value of 0.105 ng/mL for the assessment of smoking status (20).

### Statistical analyses

Under the recommendations of NHANES, all analyses incorporate MEC sample weights to make the results more representative of the entire US. Frequencies and weighted proportions were used to characterize the US patients with osteoarthritis based on CDAI concentrations, and statistical differences were tested using the Rao-Scott chi-square test. Kaplan–Meier analysis showed the difference in survival among different CDAI groups of OA patients and the significance was checked by the Logrank test. Three multivariable Cox regression models were applied to assess the relationship between CDAI and all-cause mortality and CVD mortality: Crude model was not adjusted; Model 1 was adjusted for age, gender, and ethnicity; Model 2 was adjusted for age, gender, ethnicity, education level, marital status, poverty income ratio, BMI, serum cotinine, alcohol drinking status, and history of diabetes or hypertension. In addition, in multivariate COX regressions, we performed trend tests for the quartile group of CDAI. The restricted cubic spline (RCS) regression model was conducted to investigate the linear and non-linear association of CADI with all-cause mortality and CVD mortality. We further performed an interaction test of PA and CDAI on all-cause mortality and tested the significance of multiplicative interaction terms using a likelihood ratio test. Similarly, the linear and non-linear association between CDAI and all-cause mortality at different PA levels was examined using the RCS regression.

Importantly, four sensitivity analyses were used in our current study to test the reliability of our findings. Firstly, we employed three multivariate Cox proportional risk regression models that adjusted for different confounders. Second, given the complex sampling design of NHANES, we introduced MEC weights in all analyses. Third, we conducted a stratified analysis among all covariates and performed interaction tests. Finally, after excluding individuals who died within the first two years or had cancer, we again performed three multivariate Cox proportional risk regression analyses.

All analyses were conducted with R software (version 4.2.1), and the *p* value less than 0.05 for all statistical tests in this study was considered statistically significant.

## Results

### Characteristics of the participants

**Figure 1:**
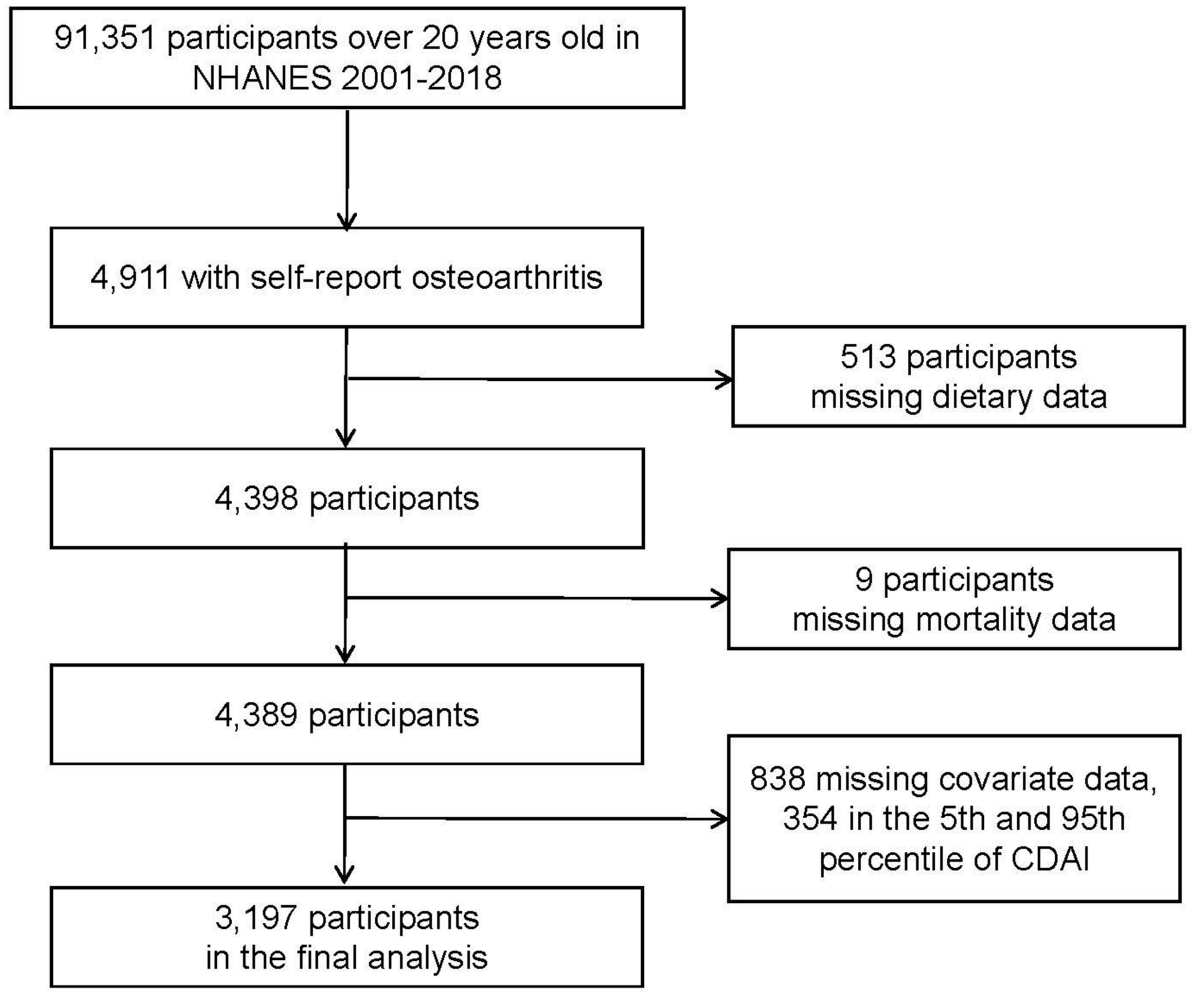
Flow chart of study.

In total 3,197 participants with OA were enrolled in the current study (Figure 1). The baseline characteristics based on CDAI concentrations are exhibited in Table 1. Significant differences in education level, marital status, poverty income ratio, serum cotinine, and alcohol drinking were found between the CDAI quartiles. For education level, the first quartile CDAI participants showed a preference for “less than high school,” whereas the fourth quartile showed a preference for “college or above” (*p* < 0.0001). The proportion of widowed/divorced/separated participants was higher in the first quartile (34.10%, *p* = 0.02). In the fourth quartile, there were more participants with higher family income-poverty ratios (53.41%, *p* < 0.0001).

**Table 1:** Characteristics of US osteoarthritis patients based on CDAI concentrations.

Characteristic	Composite dietary antioxidant index (CDAI)					*p*-value
	Total	Q1 < −2.40	Q2–2.41-0.68	Q3–0.68-1.56	Q4 > 1.56	
**Age (years), n (%)**						0.05
20–59	1,023 (40.47)	232 (37.63)	256 (40.25)	245 (37.80)	290 (45.24)	
> = 60	2,174 (59.53)	568 (62.37)	543 (59.75)	555 (62.20)	508 (54.76)	
**Gender, n (%)**						0.15
Female	2028 (64.89)	528 (69.80)	489 (63.65)	495 (63.81)	516 (63.19)	
Male	1,169 (35.11)	272 (30.20)	310 (36.35)	305 (36.19)	282 (36.81)	
**Ethnicity, n (%)**						0.11
Non-Hispanic Black	428 (5.39)	128 (7.23)	97 (4.77)	95 (4.76)	108 (5.10)	
Non-Hispanic White	2,158 (85.38)	511 (82.01)	547 (85.55)	544 (85.65)	556 (87.57)	
Mexican American	257 (2.50)	65 (2.62)	70 (2.80)	68 (2.52)	54 (2.11)	
Other Hispanic	175 (2.09)	41 (2.13)	44 (2.08)	50 (2.44)	40 (1.75)	
Other Races	179 (4.64)	55 (6.01)	41 (4.80)	43 (4.62)	40 (3.46)	
**Education level, n (%)**						**< 0.0001**
Less than high school	630 (13.11)	204 (17.24)	169 (15.45)	142 (11.48)	115 (9.40)	
High school or equivalent	761 (23.62)	209 (28.28)	195 (24.99)	165 (20.26)	192 (21.91)	
College or above	1806 (63.26)	387 (54.48)	435 (59.56)	493 (68.27)	491 (68.69)	
**Marital status, n (%)**						**0.02**
Married/cohabiting	1908 (65.30)	443 (59.38)	482 (68.83)	492 (64.97)	491 (67.03)	
Widowed/divorced/separated	1,086 (28.23)	310 (34.10)	263 (25.06)	262 (29.85)	251 (25.04)	
Never married	203 (6.47)	47 (6.53)	54 (6.11)	46 (5.18)	56 (7.92)	
**Poverty income ratio, n (%)**						**< 0.0001**
< 1.30	769 (15.33)	245 (21.44)	196 (15.72)	157 (12.00)	171 (13.34)	
1.3–3.49	1,267 (36.33)	326 (39.21)	310 (35.18)	342 (38.43)	289 (33.25)	
> = 3.50	1,161 (48.34)	229 (39.35)	293 (49.09)	301 (49.58)	338 (53.41)	
**Body mass index (kg/m2), n (%)**						0.81
< 25	650 (20.59)	166 (21.01)	155 (21.70)	167 (20.54)	162 (19.37)	
> = 25	2,547 (79.41)	634 (78.99)	644 (78.30)	633 (79.46)	636 (80.63)	
**Serum cotinine (ng/ml), n (%)**						**< 0.001**
Unexposed (< 0.105 ng/mL)	1,148 (38.89)	258 (35.03)	270 (34.95)	289 (37.21)	331 (46.80)	
Exposed(> = 0.105 ng/mL)	2049 (61.11)	542 (64.97)	529 (65.05)	511 (62.79)	467 (53.20)	
**Alcohol drinking, n (%)**						**0.001**
Non-drinker	430 (10.82)	128 (13.83)	123 (13.41)	91 (8.65)	88 (8.23)	
Drinker	2,767 (89.18)	672 (86.17)	676 (86.59)	709 (91.35)	710 (91.77)	
**Diabetes, n (%)**						0.19
No	2,524 (82.34)	607 (79.37)	619 (81.80)	651 (84.09)	647 (83.52)	
Yes	673 (17.66)	193 (20.63)	180 (18.20)	149 (15.91)	151 (16.48)	
**Hypertension, n (%)**						0.37
No	1,038 (37.88)	230 (34.00)	265 (39.29)	270 (38.84)	273 (38.73)	
Yes	2,159 (62.12)	570 (66.00)	534 (60.71)	530 (61.16)	525 (61.27)	
**Physical activity**						**0.002**
Low	1,087 (28.43)	332 (33.94)	289 (31.76)	229 (23.75)	237 (25.53)	
Insufficient	722 (22.57)	181 (24.54)	170 (20.25)	184 (22.31)	187 (23.34)	
Sufficient	1,388 (49.00)	287 (41.52)	340 (47.99)	387 (53.94)	374 (51.13)	

The bold values mean that **p** < 0.05.

### Associations of the CDAI with all-cause and CVD mortality

**Figure 2:**
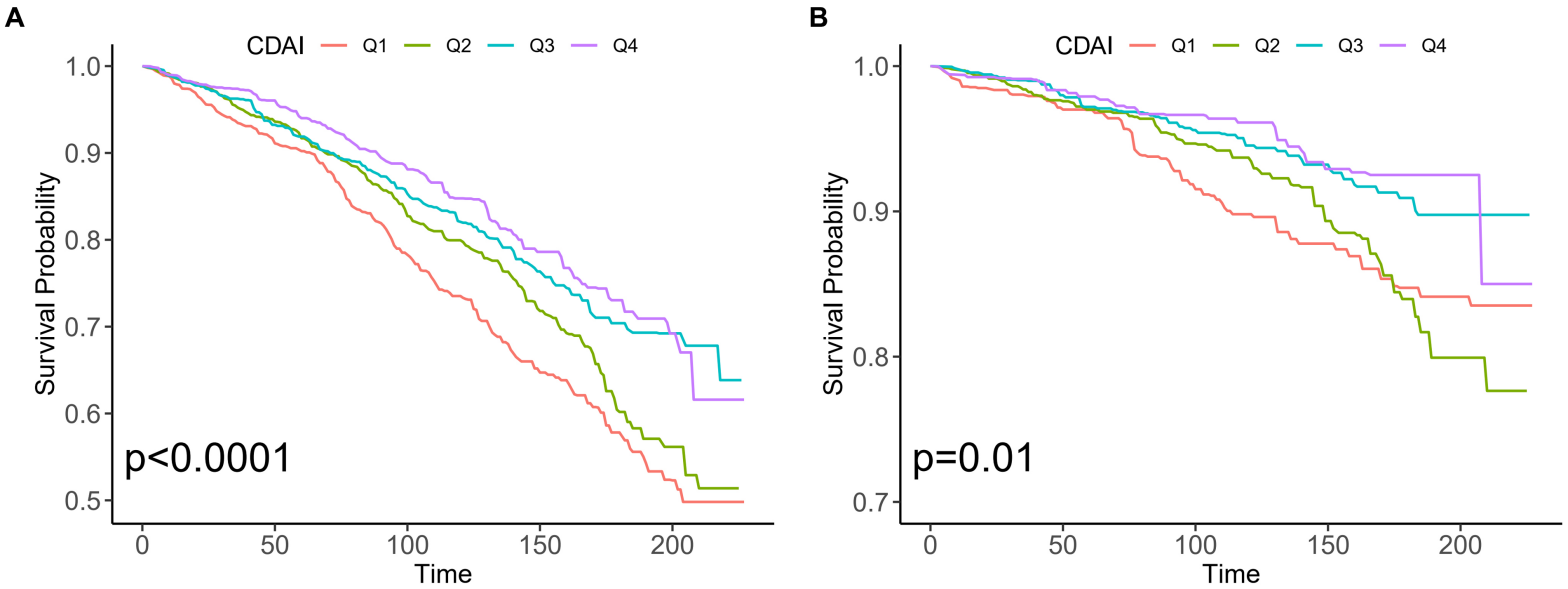
Kaplan–Meier analysis of different CDAI populations with osteoarthritis in the US. **(A)** All-cause mortality and non-mortality populations **(B)** CVD mortality and non-mortality populations. CDAI: composite dietary antioxidant index; CVD, cardiovascular disease.

During 25,564 person-years of follow-up, there were 781 all-cause deaths among 3,197 patients with OA, including 229 CVD-related deaths. As shown in Figure 2, Kaplan–Meier analysis suggested the fourth quartile (Q4) of CDAI was linked to lower all-cause mortality (*p* < 0.001) and CVD (*p* = 0.01) mortality in the early stage compared with those in the lower quartiles.

**Figure 3:**
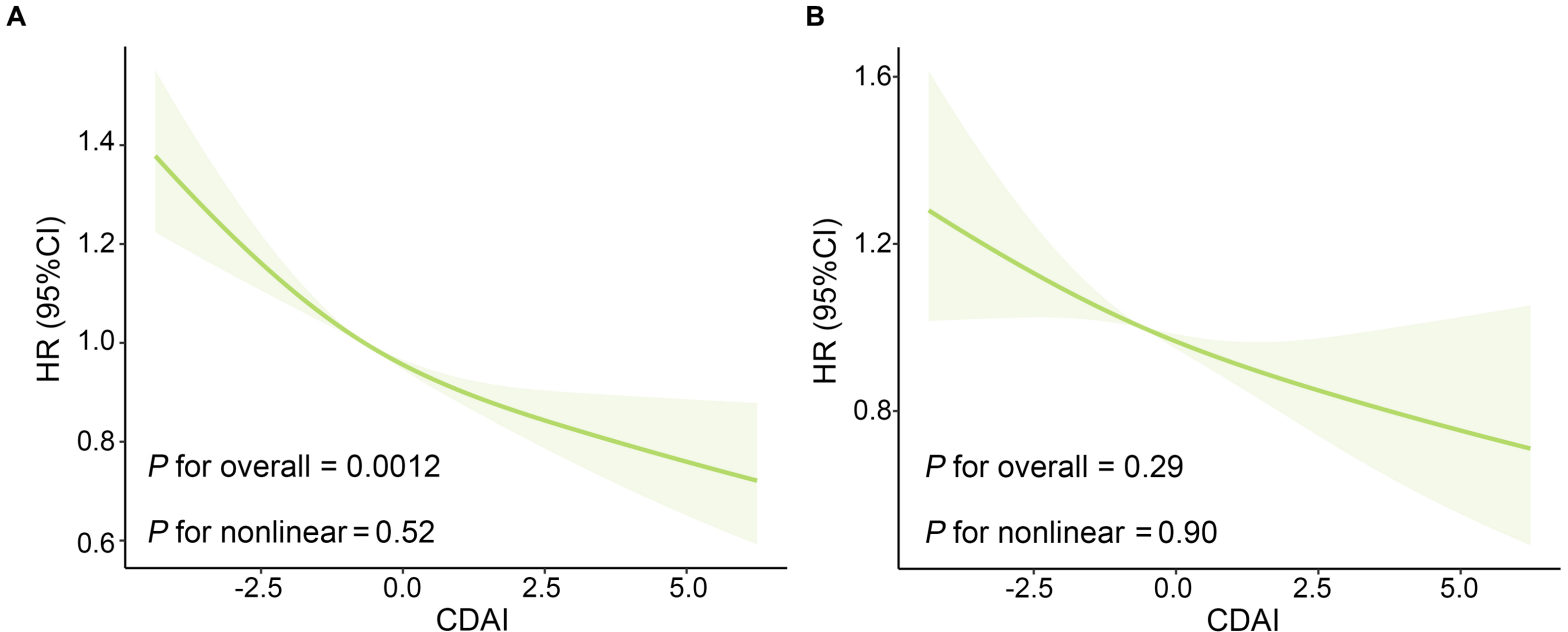
The Association of CDAI with **(A)** all-cause mortality and **(B)** CVD mortality in US patients with osteoarthritis. The solid red line is the HR value and the shadowed area is the corresponding 95% CI. The restricted cubic spline regression model was adjusted for age, gender, ethnicity, education level, marital status, poverty income ratio, BMI, serum cotinine, alcohol drinking status, and history of diabetes or hypertension. BMI, body mass index; CDAI: composite dietary antioxidant index; CVD, cardiovascular disease; CI, confidence interval; HR, hazard ratio.

Table 2 displays the Hazard ratios (HRs) with 95% confidence intervals (CIs) for all-cause and CVD mortality across CDAI quartiles. Those individuals in Q4 of CDAI had a decreased risk of all-cause mortality than those in the lower quartiles in all models. Taking the first quartile (Q1) of CDAI as reference, the weighted multivariate HRs for all-cause mortality after multivariable adjustment were 0.95 (0.77–1.17) for Q2, 0.75 (0.59–0.97) for Q3, and 0.71 (0.55–0.92) for Q4 (*P* for trend <0.001). The restricted cubic splines result in Figure 3A indicates the negative linear relationship of CDAI with all-cause mortality (*P*_0verall_ = 0.0012).

**Table 2:** Hazard ratios for all-cause and CVD mortality in US osteoarthritis patients according to CDAI.

	Composite dietary antioxidant index (CDAI)					*p* for trend
	Continuity Value	Q1	Q2	Q3	Q4	
All-cause mortality						
Crude model	0.90 (0.87, 0.93)	1.00	0.77 (0.61, 0.97)	0.64 (0.51, 0.81)	0.54 (0.42, 0.69)	**< 0.0001**
Model 1	0.91 (0.87, 0.95)	1.00	0.82 (0.66, 1.02)	0.64 (0.51, 0.82)	0.59 (0.45, 0.77)	**< 0.0001**
Model 2	0.94 (0.90, 0.97)	1.00	0.95 (0.77, 1.17)	0.75 (0.59, 0.97)	0.71 (0.55, 0.92)	**< 0.001**
CVD mortality						
Crude model	0.89 (0.81, 0.97)	1.00	0.79 (0.54, 1.15)	0.56 (0.37, 0.87)	0.45 (0.26, 0.78)	**0.002**
Model 1	0.89 (0.81, 0.97)	1.00	0.79 (0.54, 1.15)	0.51 (0.33, 0.79)	0.47 (0.28, 0.80)	**0.003**
Model 2	0.94 (0.86, 1.03)	1.00	1.00 (0.69, 1.44)	0.67 (0.42, 1.07)	0.67 (0.39, 1.13)	0.06

Crude model: No adjustment.

Model 1: Adjusted for age, Gender, Ethnicity.

Model 2: Adjusted for age, Gender, Ethnicity, Education level, marital status, poverty income ratio, BMI, serum cotinine, alcohol drinking status, and history of diabetes or hypertension.

BMI: body mass index; CDAI: composite dietary antioxidant index; CI, confidence interval; CVD, cardiovascular disease; HR, hazard ratio. The bold values mean that *p* < 0.05.

As for the association between CDAI and CVD mortality in OA patients, higher CDAI was associated with decreased risk of CVD mortality in crude model and model adjusted for age, gender and ethnicity. After adjustments for all potential confounders, there was no significant association between CDAI and CVD mortality. And subsequent restricted cubic spline (Figure 3B) reveals no non-linear association of CDAI intake with CVD mortality (*P*_overall_ = 0.29, *P*_non-linear_ = 0.90).

### Stratified and sensitivity analyses

Stratified analyses revealed that dietary CDAI had a consistent negative relationship with all-cause mortality across a variety of subgroups stratified by concomitant variables (Supplementary Table S1). Furthermore, the interaction test results revealed no detectable interaction between CDAI and stratified variables on all-cause mortality in OA populations (all *P* for interaction>0.05). Besides, the findings were broadly robust in sensitivity analyses when excluding those who died of cancer or died in the first two years of the follow-up (Supplementary Table S2).

### Interaction of PA on CDAI and all-cause mortality

**Figure 4:**
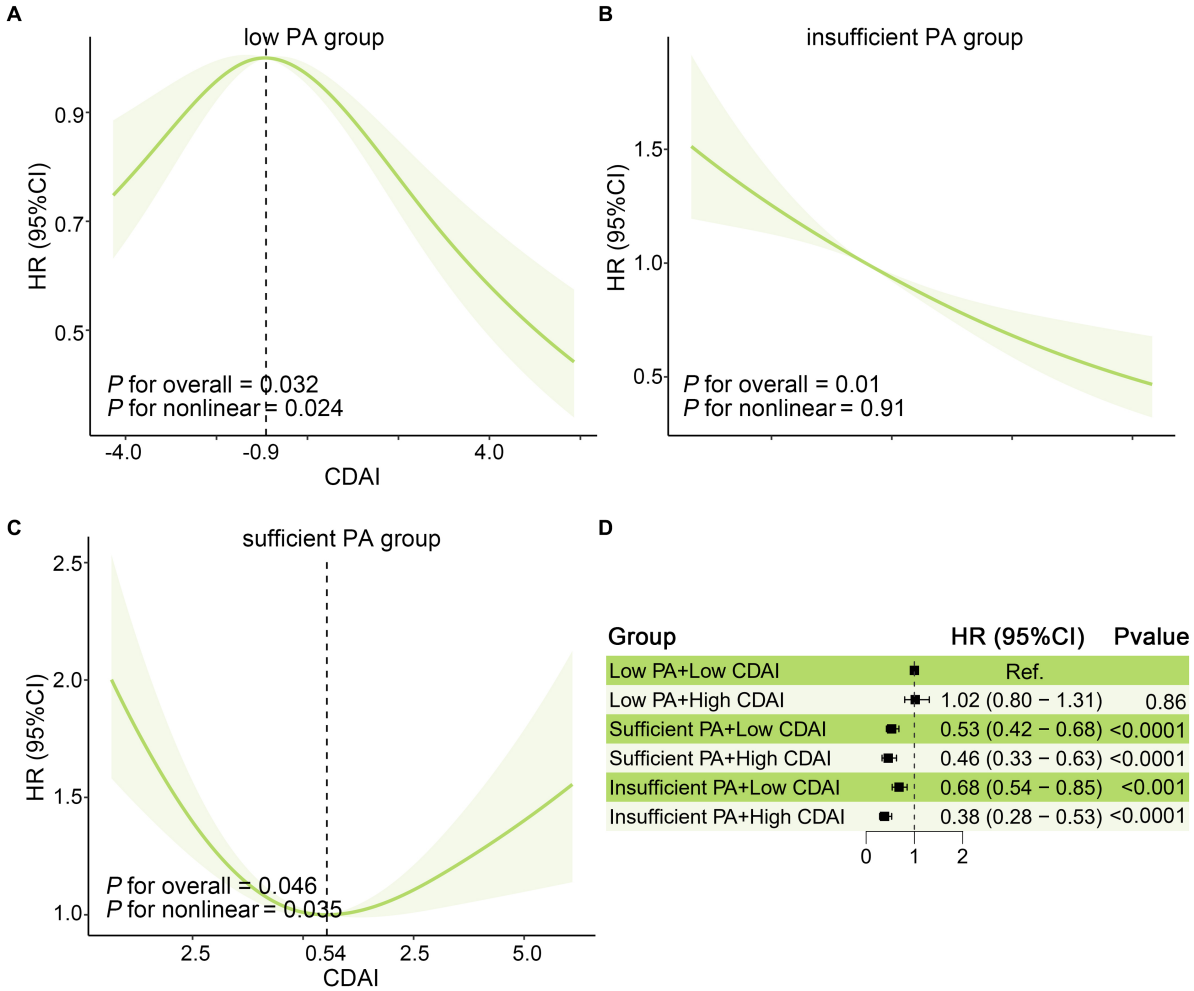
Joint affection between CDAI and PA for all-cause mortality in US patients with osteoarthritis. The association of CDAI with All-cause mortality in the US osteoarthritis population in **(A)** Low, **(B)** insufficient, and **(C)** sufficient PA groups. The solid red line is the HR value and the shadowed area is the corresponding 95% CI. **(D)** The joint effect of CDAI and PA on all-cause mortality in osteoarthritis patients. All model was adjusted for age, Gender, Ethnicity, Education level, marital status, poverty income ratio, BMI, serum cotinine, alcohol drinking status, and history of diabetes or hypertension. BMI, body mass index; CDAI: composite dietary antioxidant index; CI, confidence interval; HR, hazard ratio; PA, physical activity.

As shown in Table 3 and Supplementary Table S3, interestingly, the stratified analysis across different PA level groups suggested an interaction of PA with CDAI on all-cause mortality in OA patients (*P* for interaction = 0.01). After multivariable adjustment, higher CDAI was significantly associated with a lower risk of all-cause mortality in the insufficient PA group. In the low PA group, patients in Q1 and Q4 of CDAI had lower risk all-cause mortality compared with those in Q3 of CDAI (*P* for trend =0.23), while in the sufficient group, higher and lower CDAI were both associated with increased risk of all-cause mortality (*P* for trend =0.31). As the restricted cubic splines shown in Figures 4A–C, in the insufficient PA group, the CDAI had a negative linear relationship with all-cause mortality (*P*_overall_ = 0.01), and there were non-linear associations of CDAI with all-cause mortality in low (*P*_non-linear_ = 0.024) and sufficient PA group (*P*_non-linear_ = 0.035). HR values of all-cause mortality in the low PA group reached the highest point when CDAI = -0.90, while HR values of all-cause mortality in the sufficient PA group reached the lowest point when CDAI = 0.54. To further explore the relationship between CDAI and all-cause mortality in patients in each PA group, patients were categorized into high and low CDAI groups based on the median of CDAI. As shown in Figure 4D, taking the low PA + low CDAI group as a reference, the sufficient and insufficient PA group had lower risk of all-cause mortality, and the insufficient PA + high CDAI group had the lowest risk of all-cause mortality (HR:0.38, 95%CI: 0.28–0.53).

**Table 3:** The association of CDAI with all-cause mortality in US patients with osteoarthritis regarding different PA levels.

	Composite dietary antioxidant index (CDAI)					
	Q1 HR (95% CI)	Q2 HR (95% CI)	Q3 HR (95% CI)	Q4 HR (95% CI)	*P* for trend	*p* for interaction
**PA**						**0.01**
Low	0.82 (0.59, 1.15)	0.85 (0.61, 1.17)	1.00	**0.66 (0.45, 0.95)**	0.23	
Insufficient	1.00	0.80 (0.54, 1.19)	**0.54 (0.33, 0.88)**	**0.55 (0.35, 0.87)**	**0.002**	
Sufficient	**1.56 (1.02, 2.39)**	1.20 (0.80, 1.79)	1.00	1.17 (0.74, 1.85)	0.31	

The model was adjusted for age, gender, ethnicity, education level, marital status, poverty income ratio, BMI, serum cotinine, alcohol drinking status, and history of diabetes or hypertension.

BMI, body mass index; CDAI: composite dietary antioxidant index; CI, confidence interval; HR, hazard ratio; PA, Physical activity. The bold values mean that *p* < 0.05.

## Discussion

In this prospective cohort study based on a sizable, nationally representative sample of US OA populations, we found a negative linear association of CDAI with all-cause mortality. Besides, the further stratified analyses suggested that PA could significantly change the association between CDAI and all-cause mortality. The CDAI was negatively dose–response associated with all-cause mortality only in the insufficient PA group. The result revealed that moderate PA combined with higher CDAI could lead to decreased risk of all-cause mortality in OA patients.

The association between CDAI and mortality has been studied in several populations. According to Wang et al. in the general population, the CDAI was inversely correlated with all-cause and CVD mortality (21), which was supported by previous reports that dietary antioxidant index was associated with lower all-cause mortality in the US (22, 23), Japan (24), and Chinese populations (23). Among diabetes patients in NHANES (2003–2014), CDAI was linked to a decreased all-cause and CVD mortality (25). Similar findings were also observed in patients with stroke (11) or CKD 1–2 stages (26). Nevertheless, other studies have reported controversial findings. For example, in a prospective study among older individuals with cardiovascular risk factors, the dietary antioxidants failed to affect the mortality (27). The conflicting result could be partially explained by the small sample size, significant individual differences and brief follow-up period. From the above studies, we can speculate that the associations between CDAI and mortality in different populations are mixed. However, the evidence about CDAI and long-term health prognosis among OA patients is still limited. In the current study, OA patients with a higher CDAI were associated with a decreased risk of all-cause mortality. This finding means that OA patients should maintain higher intake of dietary antioxidants for better long-term health outcomes. Furthermore, no significant relationship between CDAI and CVD mortality was found in the OA population in the current study, larger prospective investigations are required to identify this finding.

The probable mechanisms that how CDAI affects OA patients’ mortality may be explained by oxidative stress, which is implicated in cartilage degradation and joint inflammation (6). Oxidative stress leads to telomere genomic instability, replicative senescence and dysfunction of chondrocytes, further induces cartilage homeostasis loss, which contributes to the progression of OA (5). Enhancing the body’s antioxidant capacity and reducing cartilage damage caused by oxidative stress may be a new strategy for the treatment and prevention of the occurrence and development of OA Previous studies have suggested that antioxidant nutrients such as zinc, selenium, vitamins A, C, E and carotenoids may influence the development mechanism of OA (28). Consumption of vitamin C and E may help reduce the risk of incident of knee OA (29, 30). Besides, higher consumption of vitamin C and E was associated with decreased risk of knee pain (8, 31).

Additionally, dietary santioxidants may also improve the prognosis of OA by reducing the risk of mortality of comorbidities. Chronic pain and immobility caused by OA lead to a decline in exercise ability, which results in many metabolic diseases such as obesity, diabetes, and cardiovascular disease (32). Evidence has shown that OA patients were more likely to suffer from CVD (33). A prospective study confirmed that patients with symptomatic knee OA had higher risk of CVD-specific and all-cause mortality than the healthy group (34). Besides, an increased risk for diabetes was found in people with symptomatic hip and/or knee OA (35). Many studies identified the effect of dietary antioxidants in decreasing the risk of mortality of those comorbidities. In a prospective cohort study, higher CDAI significantly reduced the risk of CVD mortality in the general population (23). Similarly, higher intake of antioxidants improved glycemic control indicators (36) and was associated with a lower risk of mortality in patients with diabetes (25). Aging, systemic chronic inflammation and immune system dysregulation are important features of OA, which are closely related to some chronic aging diseases such as CVD, obesity, type 2 diabetes mellitus and metabolic syndrome (37). Aging increases the level of ROS, inducing oxidative stress, which leads to the activation of the systemic pro-inflammatory pathway, contributing to the pathogenesis of inflammation-related diseases (37). Dietary antioxidants reduce oxidative stress, lessen systemic inflammation, and further lead to decreased risk of mortality caused by comorbidities.

In addition to diet, exercise play an crucial role in improving the prognosis of OA. As a common musculoskeletal disorder, OA causes joint pain and stiffness, resulting in decrease in physical activity and engagement (38). Much evidence suggested that moderate-intensity PA might significantly reduce mortality (39–42). A similar finding has been found in the current study of OA patients. In subgroup analysis, the result revealed that PA levels can affect all-cause mortality, the sufficient and insufficient PA group had lower all-cause mortality, than those in the low PA group. Previous randomized controlled trials and meta-analyses demonstrated PA’s effect in relieving the symptoms such as joint pain, stiffness, and activity limitations which bother the patients (43), while PA could also decrease the associated risk factors related to increased risk of death (e.g., overweight metabolic syndrome, and chronic inflammation) (12, 44). So PA was recommended as the first-line treatment for OA by many national and international guidelines. (45, 46). In our view, moderate-intensity PA might be adequate to reduce the risk of all-cause mortality in OA populations, low PA would increase the risk. However, the most effective intensity of PA in OA patients has yet to be determined.

Combined with interactive analysis, significant interaction was found between CDAI and PA on all-cause mortality. In the insufficient PA group, CDAI was negatively associated with all-cause mortality, while CDAI was non-linear associated with all-cause mortality in the sufficient and low PA group. This phenomenon can be explained by the effects of PA on oxidative stress. Previous studies reported that in skeletal muscle, PA improved antioxidant capacity, reduced pro-inflammatory and age-related oxidative stress signals, in addition, PA can also induce anabolic and activating mitochondrial biogenesis, further protecting normal cells from dysfunction (47). A dose–response relationship was described in the effects in which low levels of PA exhibit a stimulatory effect and high levels of PA exhibit an inhibitory effect (48). This means that moderate physical exercise has a synergistic effect with CDAI on oxidative stress, while high-intensity physical exercise increases oxidative stress (49), leading to a poor prognosis. Therefore, sufficient CDAI intake combined with moderate PA can significantly reduce all-cause mortality in OA patients, but for individuals, personalized diet and PA recommendations are needed to achieve greater benefits.

There are some strengths in this study. Firstly, this study focused on a nationally representative sample of the OA population in the United States, which makes it convincing to generalize the conclusions. Secondly, the National Death Index death certificate data used in long-term follow-ups to identify the death status of OA patients in this study cohort offered adequate support for the analyses in the current investigation. Furthermore, by calculating the CDAI, we assessed the overall antioxidant capacity of individuals rather than the single antioxidant diet, reducing the influence of the interaction of antioxidants on the conclusions. Additionally, we performed sensitivity and subgroup analysis to investigate the associations with consideration of numerous potential confounding variables, and the interaction of PA and CDAI on all-cause mortality in OA patients was further explained.

Nevertheless, our study also has several limitations. Firstly, the CDAI is calculated based on a 24-h dietary recall interview, which can make the estimates imprecise due to recall bias and variation in daily dietary intake. Secondly, the CDAI was calculated based on baseline antioxidant intake, typically separated from death by several years. Thus, the calculated CDAI reflects long-standing use of antioxidants, which could have changed over the long years of follow-up. Thirdly, this study fails to obtain information about the severity of OA, our findings cannot provide individualized reference for OA patients of different severities. Finally, due to the limitations of the observational study design, it is difficult to make causal inferences from the findings.

## Conclusion

Based on a nationwide representative sample of US OA populations, our findings indicate that CDAI was negatively associated with all-cause mortality, and this association was significantly changed by PA level. Higher CDAI was associated with lower risk of all-cause mortality, and this association was only observed in patients with insufficient PA. These findings suggest the potential beneficial role of higher intake of dietary antioxidants and moderate physical exercise in patients with OA. To confirm these findings, prospective, large-scale researches are required in the future.

## Data availability statement

The original contributions presented in the study are included in the article/Supplementary material, further inquiries can be directed to the corresponding author.

## Ethics statement

The protocols of NHANES were approved by the National Center for Health Statistics (NCHS) Ethics Review Board. All participants signed a written statement of consent.

## Author contributions

YZ: Formal analysis, Investigation, Writing – original draft. ZD: Methodology. HL: Writing – review & editing. GL: Formal analysis. YF: Writing – review & editing. GL: Funding acquisition. SW: Conceptualization, Validation, Writing – original draft.

## Footnotes

^1^ https://www.cdc.gov/nchs/nhanes/
