# Supplementary material for: Physical activity modifies the association of the composite dietary antioxidant index with all-cause mortality in the US osteoarthritis population
Source: Front Public Health. 2023 Dec 4;11:1297245. doi: 10.3389/fpubh.2023.1297245 (PMC10726011; doi:10.3389/fpubh.2023.1297245)
Supplement: Supplementary file 1 [file Table_1.DOCX]

Table S1. Hazard ratios for All-cause mortality in US osteoarthritis patients among different subgroups according to CDAI

| **Characteristic** | **Composite Dietary Antioxidant Index (CDAI)**  **Adjusted HR (95% CI)** | | | | ***P* for trend** | ***P* for interaction** |
| --- | --- | --- | --- | --- | --- | --- |
|  | **Q1** | **Q2** | **Q3** | **Q4** |  |  |
| **Age (years)** |  |  |  |  |  | 0.22 |
| 20-59 | 1.00 | 1.14 (0.60, 2.15) | 0.70 (0.27, 1.81) | 1.20 (0.57, 2.55) | 0.77 |  |
| > = 60 | 1.00 | 0.93 (0.75, 1.17) | 0.77 (0.59, 1.00) | 0.65 (0.49, 0.86) | <0.001 |  |
| **Gender** |  |  |  |  |  | 0.13 |
| Female | 1.00 | 0.82 (0.61, 1.10) | 0.82 (0.62, 1.10) | 0.67 (0.51, 0.88) | <0.001 |  |
| Male | 1.00 | 1.19 (0.83, 1.70) | 0.70 (0.46, 1.06) | 0.85 (0.53, 1.37) | 0.16 |  |
| **Ethnicity** |  |  |  |  |  | 0.79 |
| Non-Hispanic Black | 1.00 | 1.39 (0.76, 2.54) | 1.35 (0.73, 2.49) | 1.13 (0.58, 2.20) | 0.92 |  |
| Non-Hispanic White | 1.00 | 0.94 (0.75, 1.19) | 0.72 (0.55, 0.94) | 0.68 (0.51, 0.91) | <0.001 |  |
| Mexican American | 1.00 | 1.38 (0.38, 4.97) | 2.93 (1.24, 6.92) | 5.10 (1.69,15.32) | 0.001 |  |
| Other Hispanic | 1.00 | 0.52 (0.17, 1.59) | 0.62 (0.18, 2.14) | 0.83 (0.29, 2.40) | 0.62 |  |
| Other Race | 1.00 | 0.43 (0.07, 2.56) | 0.22 (0.03, 1.69) | 0.22 (0.02, 2.06) | 0.13 |  |
| **Body mass index (kg/m2)** |  |  |  |  |  | 0.3 |
| < 25 | 1.00 | 0.89 (0.63, 1.26) | 0.60 (0.41, 0.90) | 0.82 (0.53, 1.28) | 0.06 |  |
| > = 25 | 1.00 | 0.99 (0.79, 1.25) | 0.80 (0.59, 1.10) | 0.68 (0.50, 0.92) | 0.003 |  |
| **Serum cotinine (ng/ml)** |  |  |  |  |  | 0.58 |
| Unexposed (< 0.105 ng/mL) | 1.00 | 0.80 (0.54, 1.19) | 0.66 (0.46, 0.96) | 0.62 (0.40, 0.97) | 0.05 |  |
| Exposed (> = 0.105 ng/mL) | 1.00 | 1.01 (0.79, 1.27) | 0.83 (0.61, 1.13) | 0.76 (0.56, 1.04) | 0.01 |  |
| **Alcohol drinking** |  |  |  |  |  | 0.95 |
| Non-drinker | 1.00 | 0.78 (0.46, 1.31) | 0.68 (0.37, 1.25) | 0.60 (0.35, 1.03) | 0.08 |  |
| Drinker | 1.00 | 0.97 (0.78, 1.20) | 0.77 (0.59, 1.00) | 0.73 (0.55, 0.97) | 0.004 |  |
| **Diabetes** |  |  |  |  |  | 0.66 |
| no | 1.00 | 0.88 (0.69, 1.12) | 0.73 (0.56, 0.94) | 0.67 (0.50, 0.90) | 0.001 |  |
| yes | 1.00 | 1.13 (0.76, 1.68) | 0.88 (0.49, 1.59) | 0.83 (0.53, 1.30) | 0.21 |  |
| **Hypertension** |  |  |  |  |  | 0.11 |
| no | 1.00 | 0.97 (0.61, 1.55) | 0.47 (0.28, 0.79) | 0.64 (0.34, 1.21) | 0.01 |  |
| yes | 1.00 | 0.93 (0.73, 1.17) | 0.84 (0.63, 1.13) | 0.72 (0.53, 0.98) | 0.03 |  |

The model was adjusted for all variables except the variables themselves.

CI, confidence interval; HR, hazard ratio

Table S2. Hazard ratios for All-cause mortality by CDAI after excluding individuals with cancer and individuals who died during the first two years of follow-up.

|  | **Composite Dietary Antioxidant Index (CDAI)** | | | | | ***P* for trend** |
| --- | --- | --- | --- | --- | --- | --- |
|  | **Continuity Value** | **Q1** | **Q2** | **Q3** | **Q4** |  |
| **All-cause mortality** |  |  |  |  |  |  |
| Crude model | 0.88 (0.83, 0.92) | 1.00 | 0.77 (0.55, 1.07) | 0.57 (0.41, 0.79) | 0.49 (0.35, 0.68) | **< 0.0001** |
| Model 1 | 0.89 (0.84, 0.94) | 1.00 | 0.87 (0.64, 1.18) | 0.59 (0.43, 0.82) | 0.55 (0.39, 0.78) | **< 0.0001** |
| Model 2 | 0.92 (0.87, 0.97) | 1.00 | 1.05 (0.78, 1.42) | 0.69 (0.49, 0.98) | 0.68 (0.49, 0.95) | **0.001** |

Crude model: No adjustment

Model 1: Adjusted for age, Gender, Ethnicity

Model 2: Adjusted for age, Gender, Ethnicity, Education level, marital status, poverty income ratio, BMI, serum cotinine, alcohol drinking status, and history of diabetes or hypertension.

BMI, body mass index; CI, confidence interval; HR, hazard ratio

Table S3. Interaction test for PA and CDAI on all-cause mortality in US patients with osteoarthritis.

|  | HR (95% CI) | *P* - value | p for interaction |
| --- | --- | --- | --- |
| **PA**  Low | 0.96 (0.92, 1.01) | 0.15 | **0.04** |
| Insufficient | 0.89 (0.83, 0.95) | < 0.001 |  |
| Sufficient | 0.9 6(0.88, 1.04) | 0.31 |  |

The model was adjusted for age, gender, ethnicity, education level, marital status, poverty income ratio, BMI, serum cotinine, alcohol drinking status, and history of diabetes or hypertension.

BMI, body mass index; CDAI: composite dietary antioxidant index; CI, confidence interval; HR, hazard ratio; PA, Physical activity.
